# Removal

**Published:** 1875-09

**Authors:** Robert Battey


To my Correspondents.—The protracted illness of my wife renders it necessary that I should return to my family at Rome, Ga., where my correspondents will please address their favors. To the readers of the Journal let me say that this change will in no respect abate the deep interest which I feel in its continued prosperity and usefulness; nor will it interfere at all with the industry and zeal of my editorial labors upon it. The correspondence of the Journal should be addressed as heretofore to Atlanta. Robert Battey.

